# Dental Trauma: A Practical Guide to Diagnosis and Management

**DOI:** 10.1111/edt.12747

**Published:** 2022-03-12

**Authors:** Jilen Patel

**Affiliations:** ^1^ UWA Dental School The University of Western Australia Nedlands WA Australia; ^2^ Department of Dental Medicine Perth Children’s Hospital Nedlands WA Australia

**Keywords:** case studies, dental trauma, diagnosis, guide, management

Traumatic dental injuries (TDIs) can manifest in a multitude of permutations, occur across all age groups, involve both hard and soft tissues and carry both immediate and long‐term consequences. Moreover, these injuries can present sporadically to the dental practice and the diagnosis and management of traumatic dental injuries can often be daunting for the general dental practitioner. A new book entitled *Dental Trauma: A Practical Guide to Diagnosis and Management*, written by Serpil Djemal (Figure 1) is a compact and beautifully illustrated hardcover reference guide suitable for dental practitioners and dental students alike.^1^

**FIGURE 1:**
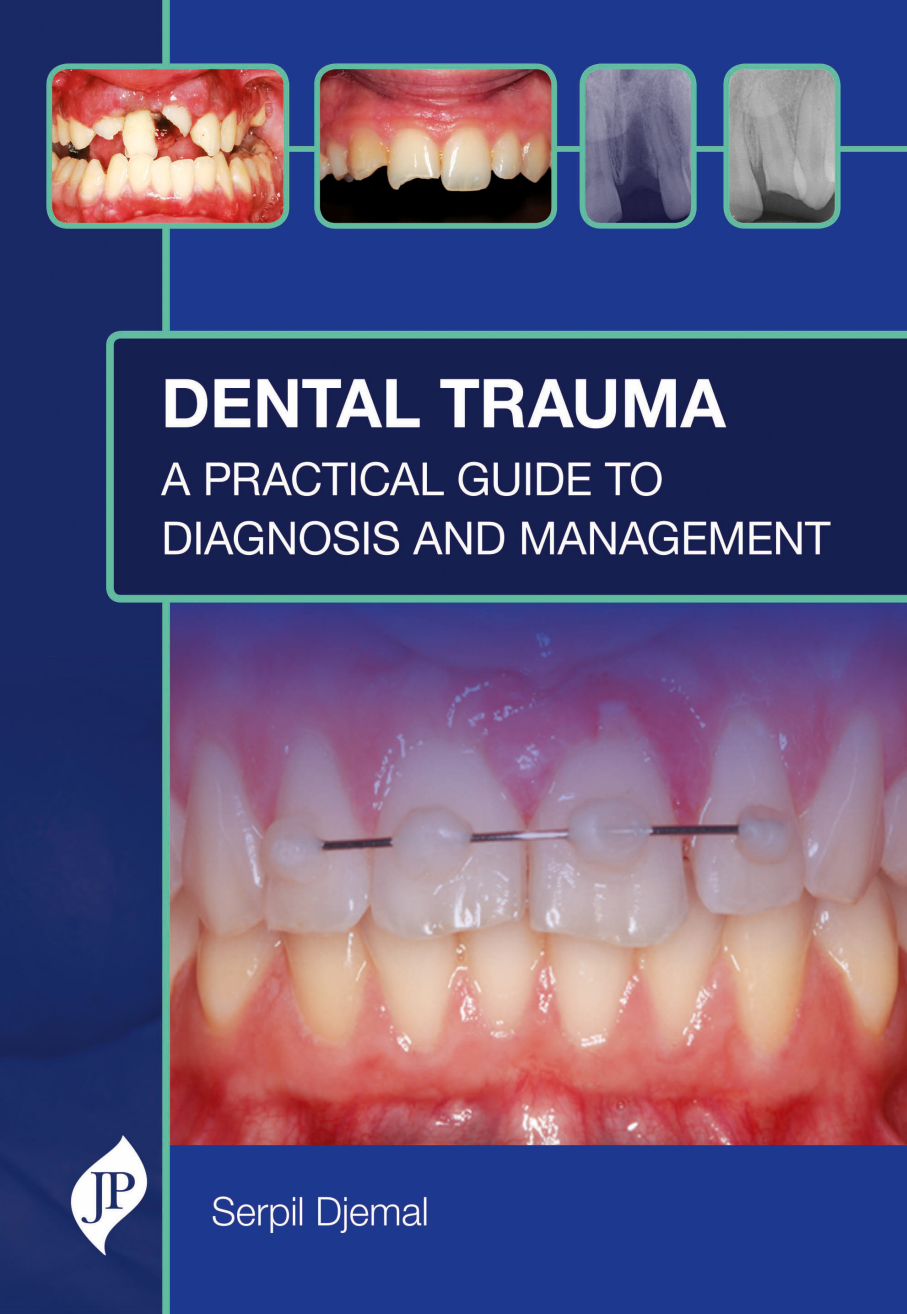
The front cover of the book

The book is systematically structured into 14 chapters classified by the type of injury arranged from fractures to luxations for easy clinical reference. Specific chapters have also been dedicated to first principles, splinting and the long‐term consequences of dental trauma. Each chapter begins with a simple two‐page summary of the TDI, followed by a series of case studies that are discussed by baseline presentation, findings of relevant imaging, diagnoses, management and follow up. Using this structure, the book does well to showcase TDIs of varying severity as well as the management of TDIs in both mature and immature permanent teeth. In keeping with the book's aim as a practical guide, each case study is comprehensively documented with high‐resolution clinical images and radiographs. These are a welcome addition for clinicians wanting to visualize the procedural steps involved in trauma management from endodontic treatment, management of soft tissues, repositioning techniques and follow‐up care.

The use of both alphanumeric and FDI notations throughout the book and within figure captions make it universally accessible to a global audience. Unique to this book, the authors have also included strategies such as mnemonics to support learning and they have presented only pertinent clinical information while limiting academic discussion to maintain clarity. Fundamental reference tables are also included in each chapter to support clinical decision making and these cover topics such as the recommended splinting times, clinical features of pulp health and features of different types of root resorption, to name a few. At the end of each chapter, relevant citations are also provided for further reading, and the most recent IADT guidelines have also been incorporated maintaining the book's contemporaneity.

The overall quality and production of this book is excellent, over 400 clinical images and radiographs have been used to make it visually striking, and the illustrations are juxtaposed with succinct, well‐structured explanations and the use of appropriate case studies. It serves as an easy to read and contemporary guide for dental trauma and a pragmatic point‐of‐care reference for dental clinicians.

## AUTHOR CONTRIBUTION

This review and the final manuscript was independently completed and written by Dr Jilen Patel.

## References

[1] Djemal S . Dental trauma: a practical guide to diagnosis and management. New Delhi, India: JP Brothers Medical Publishers; 2021.

